# A genetic ensemble approach for gene-gene interaction identification

**DOI:** 10.1186/1471-2105-11-524

**Published:** 2010-10-21

**Authors:** Pengyi Yang, Joshua WK Ho, Albert Y Zomaya, Bing B Zhou

**Affiliations:** 1 School of Information Technologies, University of Sydney, NSW 2006, Australia; 2 School of Mathematics and Statistics, University of Sydney, NSW 2006, Australia; 3 NICTA, Australian Technology Park, Eveleigh, NSW 2015, Australia; 4 Centre for Distributed and High Performance Computing, University of Sydney, NSW 2006, Australia

## Abstract

**Background:**

It has now become clear that gene-gene interactions and gene-environment interactions are ubiquitous and fundamental mechanisms for the development of complex diseases. Though a considerable effort has been put into developing statistical models and algorithmic strategies for identifying such interactions, the accurate identification of those genetic interactions has been proven to be very challenging.

**Methods:**

In this paper, we propose a new approach for identifying such gene-gene and gene-environment interactions underlying complex diseases. This is a hybrid algorithm and it combines genetic algorithm (GA) and an ensemble of classifiers (called genetic ensemble). Using this approach, the original problem of SNP interaction identification is converted into a data mining problem of combinatorial feature selection. By collecting various single nucleotide polymorphisms (SNP) subsets as well as environmental factors generated in multiple GA runs, patterns of gene-gene and gene-environment interactions can be extracted using a simple combinatorial ranking method. Also considered in this study is the idea of combining identification results obtained from multiple algorithms. A novel formula based on pairwise *double fault *is designed to quantify the degree of complementarity.

**Conclusions:**

Our simulation study demonstrates that the proposed genetic ensemble algorithm has comparable identification power to Multifactor Dimensionality Reduction (MDR) and is slightly better than Polymorphism Interaction Analysis (PIA), which are the two most popular methods for gene-gene interaction identification. More importantly, the identification results generated by using our genetic ensemble algorithm are highly complementary to those obtained by PIA and MDR. Experimental results from our simulation studies and real world data application also confirm the effectiveness of the proposed genetic ensemble algorithm, as well as the potential benefits of combining identification results from different algorithms.

## Background

It is widely acknowledged that complex diseases are most likely caused by a combination of environmental factors and interactions among multiple genes [1]. With the fast development of the genotyping technologies, single nucleotide polymorphisms (SNPs) have become one of the most commonly used biomarkers for disease associated gene identification in case-control designed genome wide association (GWA) studies [2-5]. However, there are several practical problems in analyzing the SNP genotype data. First, to identify true disease associated SNPs from a massive set of candidate SNPs, an accurate SNP selection strategy is of critical importance. However, the accurate identification of disease associated SNPs for phenotype classification is hindered by the *curse-of-dimensionality *and the *curse-of-sparsity *[6]. Furthermore, the datasets generally contain high level of data noise, high redundancy, and many missing values, and most seriously, it is evident that epistasis is a ubiquitous phenomenon in complex diseases [7,8], or in other words, gene-gene interactions and gene-environment interactions are likely to be important contributors to the development of many complex diseases (note the phrases gene-gene interaction and SNP-SNP interaction are used interchangeably in this paper). The explanations from the biological perspective are as follows: (1) a SNP in a coding region may cause amino acid substitution, leading to the functional alteration of the protein; (2) a SNP in a promoter region can affect transcriptional regulation, causing the change of the protein expression abundance; and (3) a SNP in an intron region can affect splicing and expression of the gene [9]. All these effects contribute quantitatively and qualitatively to the ubiquity of bio-molecular interactions in biological systems. Although it is a common characteristic in complex disease development, the identification of those genetic interactions have been proven to be very challenging [10].

Most of the earliest studies focused on identifying a set of SNPs in which individual SNP has a strong association with the phenotype by applying statistical measures such as *χ*^2^-statistic and logistic regression [11,12]. However, several problems arise when applying these methods. First, it is unclear how to best adjust the resulting *p*-values after testing for a very large number of possibly non-independent hypotheses. Second, complex diseases are usually caused by the action of multiple genes in a nonadditive fashion. The standard analytical approaches in GWA studies often proceed by identifying only a very small number of SNPs (usually only one or two) that exhibit strong statistical association with the target phenotype. In other words, only SNPs that independently have a strong discriminating ability are selected, while other SNPs that individually have weaker association are not discovered [13]. However, it is common that a combination of two or more SNPs, each having weak association with the phenotype, can classify the phenotypes of samples with a higher accuracy. This is natural since complex diseases are most likely caused by multiple genes and their interaction with environmental factors.

To cope with these problems, it is desirable to develop new methods which can consider multiple loci jointly. A number of such methods have been developed recently. Among these methods, nonparametric methods like Polymorphism Interaction Analysis (PIA) [14], Multifactor Dimensionality Reduction (MDR) [15], and Combinatorial Partitioning Method (CPM) [16] are the most popular ones probably due to their good generalization property on different interaction models. Specifically, PIA tries to apply multiple evaluation metrics for ranking and scoring SNP combinations while MDR and CPM attempt to modify the feature dimension to discriminate SNP-SNP interactions. However, there is no one-size-fits-all method for the detection and characterization of gene-gene interaction relationships in GWA studies. Several comparison and evaluation studies suggested that applying a combination of multiple complementary algorithms, each having its own strength, could be the most effective strategy to increase the chance of a successful analysis [10,17,18].

A recent study by McKinney *et al. *[19] proposed to use a combination of a machine learning filter and an information theoretic approach to identify SNP-SNP interactions. McKinney *et al. *found that combining the set of SNP-SNP interactions into a graph can yield interesting insights about the underlying biological processes. It is anticipated that similar network-based analysis approaches can be used as a down-stream analysis for any gene-gene interaction identification algorithms.

In this study, we attempt to address the problem from an alternative perspective by converting the issue into a combinatorial feature selection problem. From the data mining perspective, a sample from a SNP dataset of an association study is described as a SNP feature set of the form **s***_i_*={*g*_1_, *g*_2_, ..., *g_n_*}, (*i *= 1, ..., *m*) where each SNP, *g_i _*, is a categorical variable which can take the value of 0, 1, and 2 for genotypes of *aa*, *Aa*, or *AA *at this locus, and *m *is the number of samples in the dataset. The dataset can, therefore, be described as an *m *× *n *matrix **D***_mn_*={(**s**_1_, *y*_1_), (**s**_2_, *y*_2_), ..., (**s***_m_*, *y_m_*)}, where *y_i _*is the class label of the *i*^th ^sample. One can evaluate the discrimination ability of a set of SNPs jointly by applying the following two steps:

• Generating a reduced SNP feature set ${s^{\prime }}_{i}=\{g_{1},g_{2},\ldots ,g_{d}\}$, $\left({s^{\prime }}_{i}\subset s_{i}\right)$ in a combinatorial manner which restrains the dataset matrix into $D_{md}=\{({s^{\prime }}_{1},y_{1}),({s^{\prime }}_{2},y_{2}),\ldots ,({s^{\prime }}_{m},y_{m})\}$. A key observation is that feature selection algorithms which evaluate SNPs individually are not appropriate since they cannot capture the associations among multiple SNPs.

• Creating classification hypothesis *h *using an inductive algorithm, and evaluating the quality of the trained classification model using criteria such as accuracy, sensitivity, and/or specificity with an independent test set.

Without lose of generality, we use notation *s *to denote applying a SNP subset to restrain the SNP dataset **D***_mn_*. If a SNP combination *s *yields a lower misclassification rate than others, we shall consider that it possibly containing SNPs with main effects or SNP-SNP interactions with major implications. We now have two challenging problems for the SNP interaction identification. The first challenge is to generate SNP combinations efficiently since the number of SNP combinations grows exponentially with the number of SNPs and it is infeasible to evaluate all possible combinations exhaustively. The second challenge is to determine which inductive algorithm should be applied for the goodness test of SNP combinations. To tackle the first problem, we shall apply genetic algorithm (GA) since it has been demonstrated to be one of the most successful wrapper algorithms in feature selection from high-dimensional data [20,21].

Furthermore, its intrinsic ability in capturing nonlinear relationships [22] is valuable for modeling various nonadditive interactions. With regard to the second problem, there is no guiding principle on which inductive algorithms are preferable for identification of multiple loci interaction relationships. However, a promising solution is to employ an ensemble of classifiers and then to integrate/balance the evaluation results from these classifiers [23]. The key issue in applying this method is that the base classifiers used for ensemble integration should be able to capture multiple SNP interactions which commonly have nonlinear relationships. This may be achieved by using appropriate nonlinear classifiers.

The rationale of using an ensemble of classifiers can be described as follows: suppose that a given classifier *i *generates a hypothesis space ℋ*_i _*for sample classification. If the number of training samples *m *is large enough to characterize the real hypothesis *f *(in this context, *f *is the set of disease associated SNPs and SNP combinations) and the data are noise-free, the hypothesis space generated by *i *should be able to converge to *f *through training. However, since the amount of the training samples is often far too small compared to the size of the hypothesis space which increases exponentially with the size of the features (SNPs), the number of hypotheses a classifier can fit the available data is often very large. One effective way to constrain the hypothesis space is to apply multiple classifiers each with a different hypothesis generating mechanism. If each classifier fulfills the criteria of being accurate and diverse [24], it can be shown that one is able to reduce the hypothesis space to better capture the real hypothesis *f *by combining them with an appropriate integration strategy [25].

By combining GA and the ensemble of classifiers, we obtain a *genetic ensemble *(GE) algorithm for gene-gene interaction identification. The proposed algorithm has the following advantages:

• It is a nonparametric and model-free approach. Unlike traditional parametric methods (e.g. linear regression etc.), there is no need to specify and assume the number of parameters and the interaction models. As a consequence, the proposed method generalizes well and can capture a range of interaction relationship such as additive and dominant effects.

• It accommodates the detection of both linear and nonlinear relationship of gene-gene interactions. As aforementioned, the ensemble could be formed by classifiers with nonlinear separation abilities. Both linear and nonlinear gene-gene interaction relationships are common in complex disease [26], and could be captured by a nonlinear classifier [27].

• Unlike many other methods which often study different sizes of multi-loci interactions separately and repeatedly, our algorithm identifies different sizes of interactions in parallel. This feature makes the proposed algorithm particularly attractive in identifying higher-order gene-gene and gene-environment interactions.

• The system is flexible. Different inductive algorithms as well as integration methods can be readily added in for further improvements.

One other motivation for developing alternative methods for SNP-SNP interaction identification is in the hope that different algorithms may complement each other to increase the overall chance in identifying true interaction relationships. Therefore, to evaluate the degree of complementarity of multiple algorithms for SNP-SNP interaction identification is also an important part of this study. Specifically, based on the notion of *double fault *[28], we designed a formula for calculating the co-occurrence of mis-identification which gives an indication of the degree of complementarity between two different algorithms. Accordingly, the joint identification of using multiple algorithms is derived. Therefore, the contribution of this work is two-fold: (1) designing a genetic ensemble algorithm for SNP-SNP and SNP-environment interaction identification; and (2) proposing a method for evaluating the degree of complementarity among multiple algorithms.

## Methods

### Overview of genetic ensemble

In our previous study, a multi-objective GA system is implemented for high dimensional data analysis [29]. Here, we implement the GE algorithm in a similar way. The algorithm executes in an iterative manner and results collected through multiple iterations are used to assess the relative importance of SNPs and SNP-SNP combinations.

**Figure 1:**
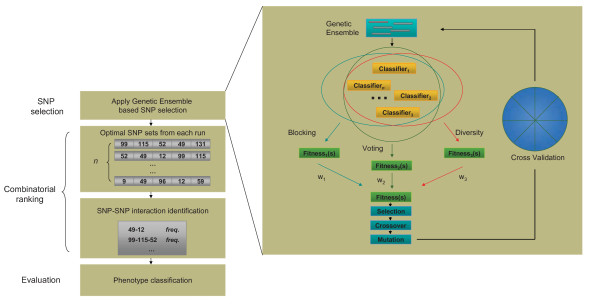
**A schematic representation of the genetic ensemble algorithm**. Multiple classifiers are integrated for gene-gene and gene-environment interaction identification. Genetic algorithm is employed to select SNP subsets that represent potential gene-gene and gene-environment interactions. After a pre-defined iterations of *n*, a total of *n *selected SNP subsets are ranked in a combinatorial manner. Each SNP combination is then assigned an identification frequency score based on the proportion of times this SNP combination is present among the *n *subsets. A SNP combination is called a functional SNP combination if it is highly ranked or if its frequency score is above a specified threshold.

As illustrated in Figure 1, the GE algorithm is applied to SNP selection repeatedly. In each run, randomly generated SNP subsets are fed into an ensemble committee for goodness evaluation. Two classifier integration strategies namely *blocking *and *voting*, and a diversity promoting method called *double fault *statistic are employed to guide the optimization process. When the evaluation of a SNP subset is completed, the feedbacks of this SNP subset are combined through a given set of weights and sent back to GA as the overall fitness of this SNP subset. After the entire population of GA is evaluated, selection, crossover and mutation are applied and the next generation begins. At the last generation of GA, the chromosome with the highest fitness is selected, and the SNP subset it represents is said to be the best SNP subset genrated by GA. The entire GA procedure is repeated (with different seeds for random initialization) *n *times (*n *= 30 in our experiments) to generate *n *best SNP subsets. These SNP subsets are then analyzed to identify frequently occurring SNP-pairs, SNP-triplets, and higher-order SNP combinations.

For SNP interaction identification, a combinatorial ranking is applied to the *n *selected SNP subsets. Each possible SNP combination is then given an identification frequency score (the number of times it appears divided by the total number of iteration *n*). For example, if the SNP combination {*snp*_1_, *snp*_2_} appears in 25 out of 30 iterations, then its identification frequency score is 25/30 = 0.833. Two alterative criteria can be used to decide whether a SNP combination should be called or not. The first criterion is to set a frequency score cut-off, say 0.8, and call all SNP combinations with frequency score higher than this cut-off as functional SNP combinations. The second criterion is to set a cut-off rank, and call all SNP combinations with equal or higher to that rank as functional SNP combinations. As will be demonstrated in subsequent sections, the choice between this two criteria is likely a balance between detection power and false discovery rate.

### Genetic algorithm

The number of SNPs considered by the genetic ensemble algorithm for potential interaction detection ranges from the lower bound of 2 to the upper bound of *d*, where *d *is the "chromosome" size of GA. The size of the GA chromosome has two implications. Firstly, it controls the number of factors we can identify. For example, if the size of *d *= 15 is used, we can identify from 2-factor up to 15-factor interactions in parallel. Secondly, *d *also influences on the size of the combinatorial space to be explored. It is a trade-off between the computational time and the combinatorial space to be searched. Therefore, for different SNP sizes (that is, the number of SNPs in the dataset), we shall use different sizes of *d *accordingly. Similar to the size of GA chromosome, the population size *p *and the generation of GA *g *are also specified according to the SNP size in the dataset. In our implementation of the GE algorithm, the parameters *d*, *p*, and g can be specified by users. The default values of these parameters are chosen empirically such they work well in a range of datasets.

For GA selection operation, we employ the tournament selection method as it gives the control of convergence speed. Specifically, the tournament selection size, denoted as *t*, is dependent on the size of the population, varying from 3 to 7. The measure for determining the winner is as follows:

(1) $$Winner=\arg\underset{s\in p}{\max}fitness(R_{i}(p))\;(i=1,2,...,t)$$

where *R_i_*(.) is the random selection function which randomly selects gene subset *s *from the GA population *p*, *t *is the tournament size, and *fitness*(.) determines the overall fitness of the randomly selected gene subset. Single point crossover is adopted with the probability of 0.7. In order to allow pair mutations, we implemented a multi-mutation strategy; that is, when a single mutation occurs (configured with the probability of 0.1) on a chromosome, another single point mutation may occur on the same chromosome with a probability of 0.25 and so on. The chromosome coding scheme is to assign an *id *to each SNP in the dataset, and to represent the chromosome as a string of SNP *id*s which specify a selected SNP subset. For each position on a chromosome, it could be a SNP *id *or a "0" which specifying an empty position.

Therefore, different sizes of SNP combinations are explored in a single GA population in parallel. Table 1 summarizes the parameter settings.

**Table 1:** Multi-objective genetic algorithm parameter settings

Parameter	Value
Genetic algorithm	Multi-objective
Chromosome size	15-25
Population size	40-340
Termination generation	8-20
Selector	Tournament selection (3-7)
Crossover	Single point (0.7)
Mutation	Multiple points (0.1 & 0.25)

The fitness of GA is defined as follows:

(2) $$fitness(s)=w_{1}\times fitness_{B}(s)+w_{2}\times fitness_{V}(s)+w_{3}\times fitness_{D}(s)$$

where *s *denotes a SNP combination under evaluation. The functions *fitness_B_*(*s*), *fitness_V _*(*s*) and *fitness_D_*(*s*) denote the fitness of a SNP combination *s *as evaluated by the *blocking*, *voting *and *double fault *diversity measures, respectively. A complexity regularization procedure is implemented in the GE algorithm to favor shorter SNP combination if two SNP combinations have the same fitness value. The computation details of each component of the fitness function are described in the next section.

### Integration strategies

#### Blocking

*Blocking *is a statistical strategy that creates similar conditions to compare random configurations in order to discriminate the real differences from differences caused by fluctuation and noise [30]. Suppose a total of *M *classification algorithms, each having a different hypothesis denoted as $h_{i}^{s}$, (*i *= 1, ..., *M*), are used to classify the data using a SNP subset *s*. The fitness function determined by *blocking *integration strategy is as follows:

(3) $$fitness_{B}(s)=\sum_{i=1}^{M}BC(p(t|h_{i}^{s},D),y)$$

where **y **is the class label vector of the test dataset **D**, function *p*(.) predicts/classifies samples in **D **as **t **using $h_{i}^{s}$, and *BC*(.) is the balanced classification accuracy devised to deal with the dataset with an imbalanced class distribution. In the binary classification, it is the area under ROC curve (AUC) [31], which can be approximated as follows:

(4) $$BC(p(t|h_{i}^{s},D),y)=\frac{Se+Sp}{2}$$

(5) $$Se=\frac{N_{TP}}{N_{case}}\times 100,\;Sp=\frac{N_{TN}}{N_{control}}\times 100$$

where *Se *is the sensitivity value calculated as the percentage of the number of true positive classification (*N_TP _*) divided by the number of cases (*N_case_*). *Sp *is the specificity value calculated as the percentage of the number of true negative classification (*N_TN_*) divided by the number of controls (*N_control_*). Such a balanced classification accuracy measure can accommodate the situation in which the dataset contains an imbalanced class distribution of cases and controls [32].

The idea of applying this strategy for classifier integration in SNP selection is that by using more classifiers to validate a SNP subset, we are able to constrain the hypothesis space to the overlap region ℋ*_o_*, increasing the chance of correctly identifying functional SNPs and SNP-SNP interactions.

#### Voting

The second classifier integration strategy applied in our GE system is *majority voting *[33]. Majority voting is one of the simplest strategies in combining classification results from an ensemble of classifiers. Despite its simplicity, the power of this strategy is comparable to many other more complex methods [34]. With a majority voting of *M *classifiers, consensus is made by *k *classifiers where:

(6) $$k\geq \{\begin{array}{ccc} M/2+1 & : & \text{if }M\text{ is}\;\text{even}\\ (M+1)/2 & : & \text{if }M\text{ is odd} \end{array}$$

Again, suppose a total of *M *classifiers, each having a different hypothesis denoted as $h_{i}^{s}$, (*i *= 1, ..., *M*), are used to classify the data using SNP subset *s*, the fitness function derived from majority voting is as follows:

(7) $$fitness_{V}(s)=BC\left(V_{k}\left(t^{\prime }|\sum_{i=1}^{M}p\text{(}t|h_{i}^{s},D)\right),y\right)$$

where **y **is the class label vector of the test dataset **D**, *V_k_*(.) is the decision function of majority voting, and **t' **is the voting prediction. Here the balanced classification accuracy *BC*(.) is calculated with voting results.

The reason for using the majority voting integration is to improve sample classification accuracy while also promoting the diversity among individual classifiers implicitly [35].

#### Double fault diversity

The third objective function is an explicit diversity promoting strategy called *double fault *statistic. This statistic is commonly used to measure the diversity of ensemble classifiers [28].

Let *c_a_*, *c_b _*∈ {*F*, *S*} in which *F *denotes the sample being misclassified by a classifier while *S *denotes the sample being correctly classified. We define $N^{c_{a}c_{b}}$ as the number of samples that are (in)correctly classified by two classifiers in which the correctness of the two classifiers is denoted by *c_a _*and *c_b _*respectively. Using this notation, we can obtain the term:

(8) $$D(p(t|h_{c_{a}}^{s},D),p(t|h_{c_{b}}^{s},D))=\frac{N^{FF}}{N}$$

which is the estimation statistic of coincident errors of a pair of classification models $h_{c_{a}}^{s}$ and $h_{c_{b}}^{s}$ (hence the name "double fault") in classification of a total of *N *samples in test dataset **D**, using SNP subset *s*.

The fitness with regard to the diversity measurement of *M *classifiers over subset *s *(denoted as *fitness_D_*(*s*)) derived from the double fault statistic are defined as follows:

(9) $$fitness_{D}(s)=1-\frac{2}{M(M-1)}\sum_{a=1}^{M}\sum_{b=a+1}^{M}D(p(t|h_{c_{a}}^{s},D),p(t|h_{c_{b}}^{s},D))$$

The value of this fitness function varies from 0 to 1. The value equals 0 when all classifiers misclassified every sample. It equals 1 when no sample is misclassified or there is a systematic diversity, leading to no sample been misclassified by any pair of classifiers.

### Classifier selection

The motivation of applying nonlinear classifiers is based on the assumption that nonlinear and nonadditive relationships are commonly presented in gene-gene interaction [26]. This is particularly relevant in detecting complex epistatic interaction that involves both additive and dominant effects. Therefore, in ensemble construction, we focus on evaluating nonlinear classifiers. Moreover, we prefer classifiers that are relatively computationally efficient since the identification of gene-gene interaction is carried out in a wrapper manner. Thus, our attention has been focused on decision tree based classifiers and instance based classifiers, as well as their hybrids because they are fast among many alternatives, while also being able to perform nonlinear classification. However, we note that any combination of linear and nonlinear classifiers can be used in our framework.

With above considerations, an initial set of experiments is conducted to select candidate classifiers for ensemble construction. The classifier selection details are presented in Results Section.

### Datasets

#### Simulation datasets

In this study, we use the simulated datasets generated by gene-gene interaction models described by Moore *et al. *[32]. In each dataset, a pair of functional SNPs which simulate gene-gene interaction are embedded along with nonfunctional SNPs, and the task is to identify this functional SNP pair from the nonfunctional ones.

For the datasets with balanced class distribution, the class ratio is 1:1 with 100 case samples and 100 control samples. The datasets are simulated under three different genetic heritability models (heritability of 0.2, 0.1, and 0.05), and two SNP sizes (SNP size of 20 and 100). This gives six sets of datasets and each set contains 100 replicates each generated with a different random seed [36]. The property of the imbalanced datasets used for evaluation is the same as the balanced datasets, except that the class ratio is approximately 1:2 with 67 case samples and 133 control samples. For imbalanced data, we restrict the evaluation to SNP size of 20, and therefore, we obtain three sets of datasets with each set containing 100 replicates. Table 2 summarizes the characteristics of the simulated datasets used for evaluation.

**Table 2:** Summary of simulation datasets

Dataset	Sample size	Ratio	Heritability	SNP size	No. replicates
balanced_200_0.2_20	200	1:1	0.2	20	100
balanced_200_0.1_20	200	1:1	0.1	20	100
balanced_200_0.05_20	200	1:1	0.05	20	100
balanced_200_0.2_100	200	1:1	0.2	100	100
balanced_200_0.1_100	200	1:1	0.1	100	100
balanced_200_0.05_100	200	1:1	0.05	100	100
imbalanced_200_0.2_20	200	1:2	0.2	20	100
imbalanced_200_0.1_20	200	1:2	0.1	20	100
imbalanced_200_0.05_20	200	1:2	0.05	20	100

#### Age-related macular degeneration dataset

Age-related macular degeneration (AMD) is the major cause of uncorrectable blindness of the elderly in many countries. As a typical complex disease, AMD is influenced by complex interactions among multiple genes and environmental factors, making it ideal for testing gene-gene interaction identification methods. In our experiment, the proposed GE algorithm, PIA, and MDR are applied to the dataset generated from a GWA study of AMD [2]. This dataset contains a genome-wide screening of 96 AMD cases and 50 controls and more than 100,000 SNPs have been genotyped for each individual.

### Evaluation statistics

#### Evaluation statistics for single algorithm

We compare the detection power of the proposed GE algorithm with PIA (version: PIA-2.0) and MDR (version: mdr-2.0_beta_6). In the previous studies of MDR [37] and PIA [14], the power of an algorithm for identifying gene-gene interactions is estimated as the percent of times the algorithm "successfully identifies" the true functional SNP pair from 100 replicates of simulated datasets. This is repeated for every heritability model to quantify how well each algorithm performs when dealing with datasets of different difficulties (lower heritability being more difficult). An algorithm is said to have successfully identified a functional SNP pair in a dataset if the true SNP-pair is reported as the top rank. For comparison with MDR and PIA, we follow this approach and estimate the power of GE, MDR, and PIA using following statistics:

(10) $$Power=\frac{N^{S}}{N}$$

where *N *is the number of datasets tested (*N *= 100 in our case), and *N^S ^*is the number of successful identification.

For GE in particular, we are also interested in estimating the distribution of false discovery rate (FDR) and true positive rate (TPR) since, in the worst case, if there is no SNP-SNP interaction in the dataset, a top-ranked interaction list only contains false positive identifications. Formally, we estimate FDR as:

(11) $$FDR(c)=\frac{N_{FP}(c)}{N(c)}$$

where *FDR*(*c*) is the FDR at the cut-off of *c*, *N_FP _*(*c*) is the number of accepted false positive identifications at the cut-off of *c*, and *N*(*c*) is the number of accepted identifications at the cut-off of *c*.

Similarly, TPR can is calculated as:

(12) $$TPR(c)=\frac{N_{TP}(c)}{N_{TP}(c)+N_{FN}(c)}$$

where *TPR*(*c*) is the TPR at the cut-off of *c*. *N_TP _*(*c*) and *N_FN _*(*c*) are the number of accepted true positives and the number of false negatives at the cut-off of *c*.

Both the rank and the identification frequency score of each SNP combination can be used as the cut-off to calculate FDR and TPR at different confidence levels. We consider both approaches and using the 100 replicate datasets of each heritability model, we obtain the average FDR and TPR at different cut-offs for each heritability model.

#### Evaluation statistics for combining algorithms

One major motivation for developing a genetic ensemble algorithm for gene-gene interaction identification is to harness the complementary strength of different classifiers such that a more robust and predictive SNP subset can be obtained. To extend this idea further, we propose to combine the inferred SNP-SNP interaction from different algorithms (such as MDR and PIA), in the hope that more robust results can be obtained. However, such benefits may come only when the results yielded by different SNP-SNP interaction identification algorithms are complementary to each other, which is analogous to the idea of the ensemble diversity.

By modifying the equation of double fault, we design the following terms to quantify the degree of complementarity (CD) of a pair of algorithms in SNP-SNP interaction identification:

(13) $$SF(X,Y)=N^{FS}+N^{SF},\;DF(X,Y)=N^{FF}$$

(14) $$CD(X,Y)=\frac{SF(X,Y)}{DF(X,Y)+SF(X,Y)}$$

where *N^XY ^*is the number of datasets with certain identification status using algorithms *X *and *Y *, and *X*, *Y *∈ {*F*, *S*} in which *F *denotes an algorithm fails to identify the functional SNP pair while *S *denotes it succeeds to identify the functional SNP pair. *SF *(*X*, *Y *) (single fault) is the number of times algorithms *X *and *Y *give inconsistent identification result, which is the situation that one algorithm succeeds while the other one fails. *DF *(*X*, *Y *) (double fault) is the number of times both *X *and *Y *fail. The pairwise degree of complementarity of the algorithms *X *and *Y *is determined by *CD*(*X*, *Y *).

Excluding the case in which both *X *and *Y *achieve 100% successful identification (which gives $\frac{0}{0}$), the value of *CD*(*X*, *Y *) varies between 0 and 1. When the results produced by *X *and *Y *are completely complementary to each other, the value of *DF *(*X*, *Y *) decreases to 0, and the value of *CD*(*X*, *Y *) reaches 1.

On the contrary, the value of *CD*(*X*, *Y *) decreases with the decrease of the degree of complementarity between algorithms *X *and *Y *, and reaches 0 when no degree of complementarity is found.

Our premise is that combining algorithms with higher degree of complementarity will yield higher identification power. In this study, we estimate the joint power of two or three algorithms as:

(15) $$Power_{J}(X,Y)=N-DF(X,Y)$$

(16) $$power_{J}(X,Y)=N-TF(X,Y,Z);\;TF(X,Y,Z)=N^{FFF}$$

where *TF *(*X*, *Y*, *Z*) is the "triple fault" which gives the coincident errors of three identification algorithms, and *Power_J _*(*X*, *Y *) and *Power_J _*(*X*, *Y*, *Z*) are the joint power of combining two and three identification algorithms respectively.

## Results

### Classifier selection and ensemble construction

**Figure 2:**
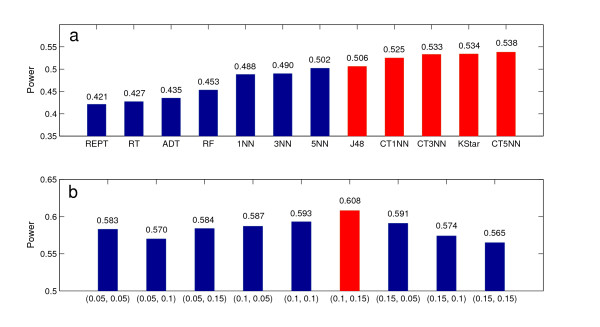
**Selection of base classifiers and ensemble configuration**. (a) Classifier selection. The value on the top of each bar denotes the estimated power in functional SNP pair identification using each classifier. (b) Ensemble configuration. The value on the top of each bar denotes the power in functional SNP pair identification using ensemble of classifiers with different values of GA chromosome mutation rate and diversity integration weight, respectively (denoted as a duplex in the X-axis).

One of the most important steps in forming an ensemble of classifiers is base classifier selection. As described above, characteristics such as nonlinear separation capability, computational efficiency, high accuracy and diversity should be taken into account. With those considerations, a classifier selection and ensemble construction experiment was carried out. Specifically, we tested the merits of each candidate classifier using datasets with model number of 10, 11, 12, 13 and 14 from Moore *et al. *[36], all of which have minor allele frequency of 0.2, heritability of 0.1, and sample size of 200 (100 case and 100 control). These are considered as "difficult" datasets since they are simulated to have low minor allele frequency, low heritability, and small sample size [14]. Twenty replicates from each model were used for evaluation and the power of each classifier in identifying the functional SNP pair was calculated. Figure 2(a) shows the 12 candidate classifiers we evaluated in this study. They are REPTree (REPT), Random Tree (RT), Alternating Decision Tree (ADT) [38], Random Forest (RT) [39], 1-Nearest Neighbor (1NN), 3-Nearest Neighbor (3NN), 5-Nearest Neighbor (5NN), Decision Tree (J48), 1-Nearest Neighbor with Cover Tree (CT1NN), 3-Nearest Neighbor with Cover Tree (CT3NN) [40], entropy based nearest neighbor (KStar) [41] and 5-Nearest Neighbor with Cover Tree (CT5NN).

The identification power of each classifier was estimated using the simulated datasets. Among the twelve classifiers, six of them successfully identified the functional SNP pair more than 50% of the time. Five of them were selected to form the ensemble (colored in red in Figure 2(a)). They are J48, KStar, and three Decision Tree and *k*-Nearest Neighbor hybrid - CT1NN, CT3NN, and CT5NN.

The configuration of parameters such as GA chromosome mutation rate and integration weights of diversity measure, blocking, and voting were tested using the same sets of data as above. Specifically, the mutation rates tested were 0.05, 0.1 and 0.15. The integration weights of diversity tested were also 0.05, 0.1 and 0.15 while the integration weights for blocking and voting were kept equal, and the three weights add up to 1. This gives 9 possible configurations for the ensemble of classifiers. The identification powers of the ensemble of classifiers using these 9 configurations are shown in Figure 2(b). It is observed that all the ensembles achieved better results than the best single classifier which has an identification power of 53.8%. Among them, the best parameter setting is (0.1, 0.15) which specifies the use of a mutation rate of 0.1 and an integration weights of 0.15, 0.425, and 0.425 for diversity, blocking, and voting, respectively. This configuration gives an identification power of 60.8% which is a significant improvement from 53.8%. This setting was then fixed in our GE in the follow up experiments.

### Simulation results

#### Gene-gene interaction identification

In the simulation experiment, we applied GE, PIA, and MDR for detecting the functional SNP pairs from 20 candidate SNPs and 100 candidate SNPs, respectively. Table 3 shows the evaluation results. By fixing the candidate SNP size to 20 and testing datasets generated with three heritability values (0.2, 0.1, and 0.05), we observed a decrease of the average identification power of the three algorithms (taking the average of the three identification algorithms) from 98.33 ± 0.94 to 78.67 ± 2.62 and to 43.67 ± 0.94. By fixing the candidate SNP size to 100 and testing datasets generated with three heritability value (0.2, 0.1 and 0.05), the average identification power drops to 93.67 ± 0.94, 48.33 ± 2.49, and 19.00 ± 1.63, respectively. It is clear that both heritability and SNP size are important factors to SNP-SNP interaction identification. By comparing the power of each algorithm, we found no significant differences. The standard deviation is generally small ranging from 0.94 to 2.62, indicating that the three algorithms have similar performance.

**Table 3:** Functional SNP pair identification in balanced datasets using GE, PIA, and MDR

Dataset	GE	PIA	MDR
	Power (%)	Power (%)	Power (%)
balanced_200_0.2_20	99	97	99
balanced_200_0.1_20	80	75	81
balanced_200_0.05_20	45	43	43
balanced_200_0.2_100	95	93	93
balanced_200_0.1_100	45	49	51
balanced_200_0.05_100	17	19	21

To investigate whether an imbalanced class distribution affects identification power, we applied GE, PIA, and MDR to imbalanced datasets with a case-control ratio of 1:2 and a candidate SNP size of 20. From Table 4, we found that the power of the three identification algorithms decreased in comparison to those of the balanced datasets (Table 3). Such a decline of power is especially significant when the heritability of the dataset is small. This finding is essentially consistent with [32] in that datasets of larger heritability values are more robust to imbalanced class distribution. Since the sample size and other dataset characteristics between the balanced and the imbalanced datasets are the same, the observed decline of power could be attributed to the imbalanced class distribution. It is also noticed that the identification power of PIA is relatively lower compared to GE and MDR. This indicates that PIA may be more sensitive to the presence of the imbalanced class distribution than GE and MDR.

**Table 4:** Functional SNP pair identification in imbalanced datasets using GE, PIA, and MDR

Dataset	GE	PIA	MDR
	Power (%)	Power (%)	Power (%)
imbalanced_200_0.2_20	92	90	95
imbalanced_200_0.1_20	59	45	62
imbalanced_200_0.05_20	32	24	27

**Figure 3:**
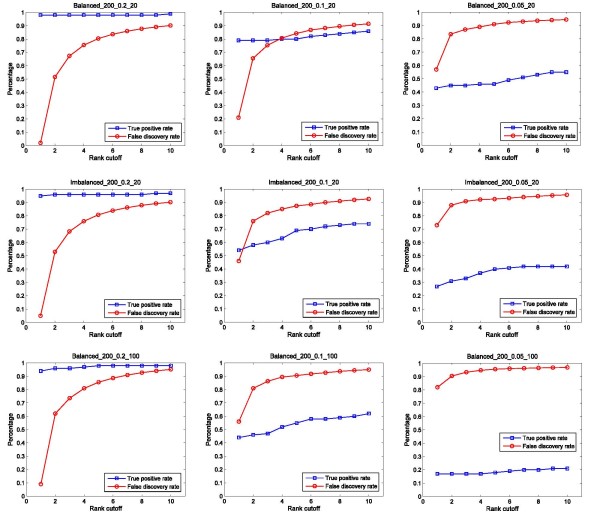
**True positive rate and false discovery rate estimation of GE at different rank cut-off**. Simulated datasets with different heritability models, number of SNPs, and class distribution, are used to evaluate the true positive rate and false discovery rate of GE at different identification cut-offs using different rank-values (1-10).

**Figure 4:**
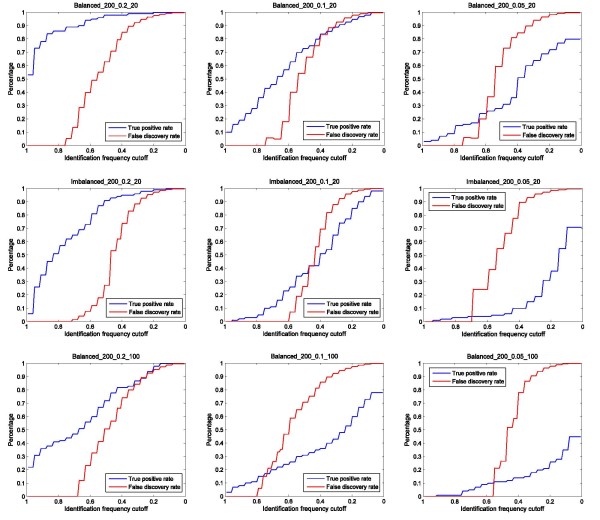
**True positive rate and false discovery rate estimation of GE at different frequency score cut-off**. Simulated datasets with different heritability models, number of SNPs, and class distribution, are used to evaluate the true positive rate and false discovery rate of GE at different identification cut-offs using different frequency scores (1-0).

For the GE algorithm, two approaches were used to study the distribution of the TPR and FDR. For the first approach, we calculated the TPR and FDR by varying the rank cut-off of the reported SNP pairs. Figure 3 shows the distribution by using a rank cut-off of 1 to 10 (the lower the number, the higher the rank). Note that the rank cut-off of 1 gives the results equal to the power defined in Equation (10). For the second approach, we calculated the TPR and FDR by varying the identification frequency cut-off of the reported SNP pairs. Figure 4 shows the distribution by decreasing the frequency cut-off from 1 to 0. By comparing the results, we found that the decrease of the heritability (from 0.2, to 0.1 and to 0.05) has the greatest impact on TPR of GE. Sample size appears to be the second factor (from 20 SNPs to 100 SNPs), and the imbalanced class distribution is the third factor (from a balanced ratio of 1:1 to an imbalanced ratio of 1:2).

Generally, by decreasing the cut-off stringency (either rank cut-off or identification frequency cutoff), the TPR increases, and therefore, more functional SNP pairs can be successfully identified. However, this is achieved by accepting increasingly more false identifications (higher FDR). The simulation results indicate that FDR calculated by using the identification frequency cut-off is very steady regardless the change of heritability, SNP size, or class ratio. In most cases, an FDR close to 0 is achieved with a cut-off greater than 0.78.

#### The degree of complementarity among GE, MDR, and PIA

As illustrated in Table 3 and Table 4, large candidate SNP size, low heritability value, and the presence of imbalanced class distribution together give the worst scenario for detecting SNP-SNP interaction. Is there a way to increase the chance of success identification in such a situation? One solution is to combine different identification results produced by different algorithms, which extends the idea of ensemble method further. However, similar to the notion of diversity in ensemble classifier, the improvement can only come if the combined results are complementary to each other. Hence, the evaluation of the degree of complementarity among each pair of algorithms becomes indispensable.

We carried out a pairwise evaluation using Equations (13) and (14). Tables 5 and 6 give the results for balanced and imbalanced situations, respectively. We observed that higher degree of complementarity is generally associated with higher identification power. For the balanced datasets, the degree of complementarity of PIA and MDR is relatively low compared to those generated by GE and PIA, or GE and MDR. The results indicate that the GE algorithm, which tackles the problem from a different perspective, is useful in complementing methods like PIA and MDR in gene-gene interaction identification. As for the imbalanced datasets, the difference of the complementarity degree between each pair of algorithms is reduced. This suggests that more methods need to be combined for imbalanced datasets in order to improve identification power.

**Table 5:** Functional SNP pair identification in balanced datasets by combining multiple algorithms

Dataset	(GE + PIA)		(GE + MDR)		(PIA + MDR)		(GE + PIA + MDR)
	CD	Power_J _(%)	CD	Power_J _(%)	CD	Power_J _(%)	Power_J _(%)
balanced_200_0.2_20	1000	100	1.000	100	0.667	99	100
balanced _200_0.1_20	0.448	84	0.556	88	0.240	81	88
balanced_200_0.05_20	0.303	54	0.303	54	0.068	45	55
balanced_200_0.2_100	1.000	100	0.923	99	0.444	95	100
balanced_200_0.1_100	0.441	62	0.400	61	0.148	54	63
balanced_200_0.05_100	0.093	22	0.116	24	0.025	21	24

**Table 6:** Functional SNP pair identification in imbalanced datasets by combining multiple algorithms

Dataset	(GE + PIA)		(GE + MDR)		(PIA + MDR)		(GE + PIA + MDR)
	CD	Power_J _(%)	CD	Power_J _(%)	CD	Power_J _(%)	Power_J _(%)
imbalanced_200_0.2_20	0.714	96	0.818	98	0.750	97	99
imbalanced_200_0.1_20	0.567	71	0.481	73	0.475	68	76
imbalanced_200_0.05_20	0.286	40	0.301	42	0.287	38	47

**Figure 5:**
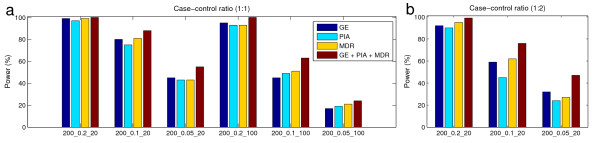
**A comparison of identification power of GE, PIA, MDR, and combination of the three algorithms**. The name of each dataset denotes sample size, heritability, and the number of SNP (SNP size). (a) Identification power of each algorithm and their joint power using datasets with balanced class distribution. (b) Identification power of each algorithm and their joint power using datasets with imbalanced class distribution.

The last columns of Tables 5 and 6 show the joint identification power of the three algorithms in analyzing balanced and imbalanced data. These results indicate a significant recovery of detection ability in functional SNP pair identification by applying three algorithms collaboratively. This is especially true when analyzing imbalanced datasets and the heritability of the underlying genetic model is low. For example, the average identification power of three algorithms for imbalanced datasets with heritability of 0.1 and 0.05 are 55.33% and 27.67%, respectively (Table 4). By combining the results of the three algorithms, we are able to increase the power to 76% and 47%, respectively, improving by around 20% (Figure 5).

### Real-world data application

As an example of real-world data application, we applied the GE algorithm, PIA and MDR, to analyze the complex disease of AMD. To reduce the combinatorial search space, we followed the two-step analysis approach [42] and used a SNP filtering procedure that is similar to the method described in [23] which can be summarized as follows:

• Excluding SNPs which have more than 20% missing genotype values of total samples;

• Calculating allelic *χ*^2^-statistics of each remaining SNP and keeping SNPs which have a *p*-value smaller than 0.05 while discarding others. A number of 3583 SNPs passed filtering.

• Utilizing RTREE program [43] to select top splitting SNPs in AMD classification. Two SNPs with *id *of rs380390 and rs10272438 are selected.

• Utilizing Haploview program [44] to construct the Linkage Disequilibrium (LD) blocks around above two SNPs.

After the above processing steps, we obtained 17 SNPs from the two LD blocks. They are rs2019727, rs10489456, rs3753396, rs380390, rs2284664, and rs1329428 from the first block, and rs4723261, rs764127, rs10486519, rs964707, rs10254116, rs10486521, rs10272438, rs10486523, 10486524, rs10486525, and rs1420150 from the second block. Based on the previous investigation of AMD [45-47], we added another six SNPs to avoid analysis bias. They are rs800292, rs1061170, rs1065489, rs1049024, rs2736911, and rs10490924. Moreover, environment factors of Smoking status and Sex are also included for potential environment interaction detection. Together, we formed a dataset with 25 factors for AMD association screening and gene-gene interaction identification.

Tables 7 and 8 illustrate the top 5 most frequently identified 2-factor interactions and 3-factor interactions, respectively. At the first glance, we see that the identification results given by different methods are quite different from one another. Considering the results of 2-factor and 3-factor interaction together, however, we find that two gene-gene interactions and a gene-environment interaction are identified by all three methods. Specifically, the first gene-gene interaction is characterized by the SNP-SNP interaction pair of rs10272438×rs380390. The first SNP is a A/G variant located in intron 5 of *BBS9 *located in 7p14, while the second SNP is a C/G variant located in intron 15 of *CFH *located in 1q32. The second frequently identified gene-gene interaction is characterized by the SNP-SNP interaction pair of rs10490924×rs10272438. The first SNP in this interaction pair is a nonsynonymous coding SNP of Ser69Ala alteration located in exon 1 of *ARMS2 *located in 10q26. And the second SNP is again the A/G variant located in intron 5 of *BBS9 *located in 7p14. As to the gene-environment interaction pair, it is characterized by rs10272438×Sex. This pair indicates that SNP factor of the A/G variant located in intron 5 of *BBS9 *in location of 7p14 is likely to associate with the disease differently between male and female.

**Table 7:** Two-factor interaction candidates of AMD dataset

GE	CV Acc %	PIA	CV Acc %	MDR	CV Acc %
rs10272438×rs4723261	68.5	**rs10272438**×**rs380390**	64.2	rs10490924×rs1420150	65.5
rs10272438×rs2736911	66.9	**rs10490924**×**rs10272438**	68.2	rs10272438×rs1065489	68.4
rs10272438×rs964707	68.5	Y402H×rs10272438	65.5	rs10272438×rs2284664	66.7
**rs10272438**×**Sex**	67.5	rs10254116×Smoking	67.1	**rs10272438**×**Sex**	67.5
rs10272438×rs2284664	66.7	rs10490924×rs10254116	67.7	rs10254116×rs2736911	67.7

**Table 8:** Three-factor interaction candidates of AMD dataset

GE	CV Acc %	PIA	CV Acc %	MDR	CV Acc %
rs10272438×rs4723261×rs964707	68.5	**rs10272438×rs380390×**rs10486524	59.8	**rs10272438×rs380390×**rs10486524	59.8
rs10272438×rs4723261×rs2736911	67.1	**rs10272438×rs380390×Sex**	61.2	**rs10272438×rs380390×**rs964707	63.4
**rs10272438×rs380390×**rs964707	63.4	**rs10272438×Sex×**rs1065489	68.1	Y402H×rs10272438×rs964707	60.7
**rs10490924×rs10272438**×rs4723261	65.0	**rs10272438×rs380390**×rs10254116	66.6	**rs10490924×rs10272438×Sex**	63.4
**rs10272438×Sex**×rs4723261	63.5	**rs10272438×rs380390**×rs1420150	59.4	**rs10272438×Sex**×rs2736911	65.7

We also test the association of Age factor with AMD by using Gaussian discretization to partition the age value of each sample into three categories as follows:

(17) $$age(x)=\{\begin{array}{ll} “\text{young}” & x\leq \mu -\sigma /2\\ \begin{array}{l} “\text{medium}”\\ “\text{elderly}” \end{array} & \begin{array}{l} \mu -\sigma /2<x<\mu +\sigma /2\\ x\geq \mu +\sigma /2 \end{array} \end{array}$$

where *μ *is the average age value and *σ *is the standard deviation of age values.

After including the Age factor to the dataset, all three algorithms identified the gene-environment interaction of rs1420150×Age as the interaction with major implication, indicating Age factor is, expectedly, strongly associated with the development of AMD. The SNP that interacted with the Age factor is a C/G variant located in intron 9 of *BBS9 *located in 7p14.

Table 9 summarizes the factors involved in potential interactions identified by all of the three different algorithms. Overall, the experimental results suggest that genes of *BBS9 *(Bardet-Biedl syndrome 9), *CFH *(complement factor H), and *ARMS2 *(age-related maculopathy susceptibility 2) with the external factors of Age and Sex, and the interactions among them are strongly associated with the development of AMD. This is essentially consistent with current knowledge of AMD development in the literature [2,45-47].

**Table 9:** SNPs and environmental factors strongly associated with AMD

Factor	Chrom.	Gene	Location	Effect	Main effect *p*-value
rs10272438	7p14	*BBS9*	intron 5	A/G	1.4 × 10^6^
rs1420150	7p14	*BBS9*	intron 9	C/G	2.1 × 10^-2^
rs380390	1q32	*CFH*	intron 15	C/G	4.1 × 10^-8^
rs10490924	10q26	*ARMS2*	exon 1	Ser69Ala	1.8 × 10^-3^
Sex	-	-	-	-	1.4 × 10^-2^
Age	-	-	-	-	1.1 × 10^-3^

## Discussion and conclusion

How multiple genes contribute to the development of complex diseases is an essential question for complex disease study. This is because a single gene often does not have the power to discriminate the status of the complex disease, and it is likely that multiple genes each with a weak or moderate effect together contribute to the development of complex disease. Although great effort has been devoted to characterizing such gene-gene interactions in complex disease analysis, the results remain unsatisfactory.

The advance of high-throughput genotyping technologies provides the opportunity to elucidate the mechanism of gene-gene and gene-environment interaction via SNP markers. However, current algorithms have limited power in terms of identifying true SNP-SNP interactions. Moreover, the simulation results indicate that the factors such as heritability, candidate SNP size, and the presence of imbalanced class distribution all have profound impact on a given algorithm's power in identifying functional SNP interactions. One practical way to improve the chance of identifying SNP-SNP interactions is to combine different methods where each addresses the same problem from a different perspective. The rationale is that the consensus may increase the confidence of identifications and complementary results may improve the power of identification.

Due to these considerations, we proposed a hybrid algorithm using genetic ensemble approach. Using this approach, the problem of SNP-SNP interaction is converted to a combinatorial feature selection problem. Our simulation study indicates that the proposed GE algorithm is comparable to PIA and MDR in terms of identifying gene-gene interaction for complex disease analysis. Furthermore, the experimental results demonstrate that the proposed algorithm has a high degree of complementarity to PIA and MDR, suggesting the combination of GE with PIA and MDR will likely lead to higher identification power.

For the practical application of the GE algorithm, the experimental results from the simulation datasets suggest that taking the top-ranked result generally gives a higher sensitivity of identifying SNP-SNP interactions than using a frequency score cut-off. However, if the delectability of the SNP-SNP interaction is low or no such interaction is present in the dataset, the top-ranked result is likely to be a false positive identification. A more conservative approach is to use an identification frequency cut-off of 0.75-0.8 which in our simulation study gives identification results with an FDR close to 0. For any identified SNP pair with an identification frequency higher than 0.8, the confidence is very high.

As a down-stream analysis, we can fit the identified SNP pairs using logistic model with interaction terms and calculate the *p*-value of its coefficient in order to quantify the strength of the interaction. In particular, to test additive and dominant effects, we can fit the reported SNP combinations using the model described by Cordell [12] and analyze the coefficients associated with additive and dominant effects of each SNP.

Current GWA studies commonly produce several hundreds of thousands of SNPs, yet the gene-gene interaction identification algorithms like MDR, PIA and the proposed GE algorithm can only cope with a relatively small number of SNPs in a combinatorial manner. Therefore, a filtering procedure is required to reduce the number of SNPs to a "workable" amount before those combinatorial methods can be applied to datasets generated by GWA studies [48,49]. More efforts are required to seamlessly connect these two components to maximize the chance of detecting complex interactions among multiple genes and environmental factors [42].

In conclusion, we proposed a GE algorithm for gene-gene and gene-environment interaction identification. It is comparable to two other state-of-the-art algorithms (PIA and MDR) in terms of SNP-SNP interaction identification. The experimental results also demonstrated the effectiveness and the necessity of applying multiple methods each with different strengths to the gene-gene and gene-environment interaction identification for complex disease analysis.

## Authors' contributions

PY conceived the study, designed, and implemented the algorithms, and performed the experiments. PY and JWKH interpreted the results and drafted the manuscript. BZ and AYZ revised the manuscript critically and supervised research. All authors read and approved the final manuscript.

## Availability

The GE algorithm (GEsnpx) is implemented in Java. It is freely available from the supplementary website at http://www.cs.usyd.edu.au/~yangpy/software/GEsnpx.html
